# Endurance performance is enhanced by intermittent hyperbaric exposure via up‐regulation of proteins involved in mitochondrial biogenesis in mice

**DOI:** 10.14814/phy2.13349

**Published:** 2017-08-04

**Authors:** Junichi Suzuki

**Affiliations:** ^1^ Laboratory of Exercise Physiology Health and Sports Sciences Course of Sports Education Department of Education Hokkaido University of Education Midorigaoka Iwamizawa Hokkaido Japan

**Keywords:** Exercise training, intermittent hyperbaric exposure, metabolic enzymes, PGC‐1*α*

## Abstract

This study was designed to (1) investigate the effects of acute hyperbaric exposure on muscle mRNA expression levels, and (2) clarify the mechanisms by which intermittent hyperbaric exposure improves endurance capacity. Experiment 1: Male mice were subjected to acute 1‐h hyperbaric exposure (1.3 atmospheres absolute with 20.9% O_2_). The expression of peroxisome proliferator‐activated receptor gamma coactivator 1‐alpha (PGC‐1*α*) mRNA significantly increased in the soleus (7.2‐fold) and red gastrocnemius muscles (Gr, 5.1‐fold) 3 h after hyperbaric exposure. Peroxisome proliferator‐activated receptor alpha (PPAR *α*) mRNA levels significantly increased in the plantaris (PL, 2.9‐fold) and Gr (2.3‐fold) 3 h after hyperbaric exposure. Experiment 2: Mice were subjected to exercise training with (HypTr) and without (Tr) 1‐h hyperbaric exposure for 4 weeks. Increases in maximal exercise capacity were significantly greater in HypTr than in Tr. In PL, activity levels of 3‐hydroxyacyl‐CoA‐dehydrogenase and citrate synthase (CS) were significantly greater in HypTr than in Tr. CS and phosphofructokinase activities both markedly increased in the white gastrocnemius muscle (Gw) in HypTr only. PGC‐1*α* expression in the nucleus was significantly greater in HypTr than in Tr in PL (4.8‐fold), Gr (3.2‐fold), and Gw (15‐fold). Protein levels of mitochondrial transcription factor A and heat shock protein 70 significantly increased after training with hyperbaric exposure. These results suggest that exercise training with intermittent hyperbaric exposure represents a beneficial strategy for increasing endurance performance by facilitating oxidative and glycolytic capacities and the expression of proteins involved in mitochondrial biogenesis in the hindlimb muscles.

## Introduction

Chronic exposure to hyperbaric oxygen has been shown to facilitate the healing of injuries such as bone fracture (Kawada et al. 2013) and muscle injury (Best et al. 1996; Asano et al. 2007). However, its ability to improve athletic performance has not yet been established.

A performance test following acute exposure to 2.0 atmospheres absolute (ATA) with 100% O_2_ for 1 h revealed that neither oxygen consumption nor endurance performance was facilitated in humans (Webster et al. 1998). In trained endurance athletes, an exercise performance test following acute hyperbaric exposure at 2.5 ATA with 95% O_2_ for 90 min revealed no enhancements in submaximal or maximal exercise performance (McGavock et al. 1999).

In contrast to hyperbaric exposure in a hospital or laboratory, a commercial hyperbaric apparatus functions at <1.5 ATA with room air (20.9% O_2_). Although this commercial product has become popular in the field of sports, its beneficial effects of improving exercise performance remain controversial.

Uninterrupted hyperbaric exposure (1.25 ATA) with 20.9% O_2_ for 7 days facilitated regeneration after severe muscle injuries in rats (Fujita et al. 2014). When hyperbaric exposure is applied to daily training regimens for human athletes, the duration of exposure needs to be as short as possible to alleviate physical and mental stresses and not affect routine training regimes. However, intermittent hyperbaric exposure (<1.5 ATA with 20.9% O_2_) for approximately 1 h per day has not been experimentally investigated. Thus, this study was performed to clarify whether acute hyperbaric exposure (1.3 ATA with 20.9% O_2_) alters gene expression and if chronic intermittent exposure induces the expression of proteins involved in mitochondrial biogenesis in mice.

Peroxisome proliferator‐activated receptor gamma coactivator 1‐alpha (PGC‐1*α*) has been implicated in the regulation of skeletal muscle adaptation in response to exercise training (Wu et al. 1999; Vega et al. 2000). PGC‐1*α* was shown to up‐regulate the transcription of mitochondrial transcription factor A (Tfam) (Wu et al. 1999). In response to muscle contraction, PGC‐1*α* translocates to mitochondria and forms a complex with Tfam, thereby facilitating mitochondrial biogenesis through the transcription of mitochondrial genome‐encoded genes and mtDNA replication. The content of the PGC‐1*α*‐Tfam complex in muscle mitochondria was shown to be markedly increased immediately and 3 h after acute exhaustive exercise in mice (Safdar et al. 2011) It currently remains unclear whether chronic exercise training with intermittent hyperbaric exposure affects PGC‐1*α* and Tfam protein expression in skeletal muscle.

In this study, experiments were designed to (1) investigate the effects of acute intermittent hyperbaric exposure on muscle mRNA expression levels, and (2) elucidate the effects of exercise training with intermittent hyperbaric exposure on endurance capacity as well as muscle metabolic capacity and the expression of proteins involved in mitochondrial biogenesis in mice.

## Materials and Methods

### Ethical approval

All procedures were approved by the Animal Care and Use Committee of Hokkaido University of Education and performed in accordance with the “Guiding Principles for the Care and Use of Animals in the Field of Physiological Sciences” of the Physiological Society of Japan and the “European Convention for the Protection of Vertebrate Animals used for Experimental and other Scientific Purposes” (Council of Europe No. 123, Strasbourg 1985).

### Experiment 1

#### Animals and acute hyperbaric exposure

Thirty male ICR (MCH) mice (10 weeks old) were purchased from Clea Japan Inc. (Tokyo, Japan) and housed under the conditions of a controlled temperature (24 ± 1°C) and relative humidity of approximately 50%. Lighting (7:00–19:00) was controlled automatically. All mice were given commercial laboratory chow (solid CE‐2, Clea Japan) and tap water ad libitum. After mice had been fed for 2 weeks and allowed to adapt to the new environment, they were randomly assigned to a sedentary control group (Sed, *n* = 6) and hyperbaric exposure group (*n* = 24). The hyperbaric exposure group was subjected to 1.3 ATA with room air (20.9% O_2_) for 1 h using HBA Space 72H (Nippon Light Service Inc., Tokyo, Japan). This 1‐h exposure included a pressurizing phase for 6 min (0.05 ATA min^−1^) and depressurizing phase for 4 min (0.075 ATA min^−1^). Following the exposure, tissues were collected at either 0 (immediately after the treatment, *n* = 6), 3 (*n* = 6), 6 (*n* = 6), or 10 (*n* = 6) h. Mice were anesthetized with *α*‐chloralose (0.06 g kg^−1^ i.p.) and urethane (0.7 g kg^−1^ i.p.). A toe pinch response was used to validate adequate anesthesia. The soleus (SOL), plantaris (PL), and gastrocnemius muscles were excised and the deep red region (Gr) of the gastrocnemius was isolated from the superficial white region (Gw). All muscles were frozen in liquid nitrogen for biochemical analyses. Mice were killed by excision of the heart. The heart was weighed and frozen in liquid nitrogen. All tissue samples were stored at −80°C until later analyses.

#### Real‐time PCR analyses

Total RNA was isolated using RNAzol RT (Molecular Research Center, Inc., OH). To assess mRNA, 2 *μ*g of total RNA was taken for cDNA synthesis using an oligo dT primer and Mmlv reverse transcriptase (RNase H minus point mutant, ReverTra Ace, Toyobo Co., Tokyo, Japan).

mRNA levels were measured by a standard real‐time polymerase chain reaction using the KAPA SYBR FAST qPCR Kit (KAPA Biosystems Inc., MA). Both retention in endoplasmic reticulum‐1 (Rer‐1) and hypoxanthine ribosyltransferase (HPRT) were used as endogenous controls for mRNA expression analyses. The PCR conditions employed for mRNA were: pre‐denaturation at 95°C for 1 min, and then denaturation at 95°C for 10 sec, annealing at 60 or 62°C for 20 sec, and extension at 70°C for 1 sec for 40 cycles. A real‐time analysis of PCR amplification was performed on a CFX96 real‐time PCR system and analyzed with the CFX Manager software (Bio‐Rad Inc., CA). Serial 5‐fold dilutions of a cDNA sample were used to generate a standard curve. Nonspecific products such as primer dimer formation were examined using dissociation curves and the results of negative control samples without cDNA. The sequences of the forward and reverse primer sets were as follows: Rer‐1 (GenBank accession number NM‐026395.1, forward: 5′‐ACCGGAGCTGCGAGTTACAGAA‐3′, reverse: 5′‐ TAGACTTGTCCAGCCAGGACTGA‐3′); HPRT (NM_013556.2, forward: 5′‐CGACCCTCAGTCCCAGCGTCGTGATTA‐3′, reverse: 5′‐AGGGCCACAATGTGATGGCCTCCCA‐3′); PGC‐1*α* (NM_008904.2, forward: 5′‐ TCCTCACACCAAACCCACAGA‐3′, reverse: 5′‐AACCCTTGGGGTCATTTGGTGA‐3′); PPAR*α* (NM_011144.6, forward: 5′‐TGCATTTGGGCGTATCTCACC‐3′, reverse: 5′‐CAGAGCGCTAAGCTGTGATGA‐3′).

### Experiment 2

#### Animals, hyperbaric exposure, and exercise training

Thirty‐seven male ICR (MCH) mice (10 week old) were purchased and housed as described above. After mice had been fed for 2 weeks and allowed to adapt to the new environment, they were randomly assigned to a sedentary control group (Sed, *n* = 9), intermittent hyperbaric exposure group (Hyp, *n* = 8), and training group (*n* = 20). To familiarise mice with the treadmill device, mice in the training group were subjected to treadmill walking three times a week during the second week of the acclimation period using a controlled treadmill (Modular motor assay, Columbus Instruments Inc., Columbus, OH) for 3 min per day at 10–15 m min^−1^ with a 5 deg incline.

**Figure 1:**
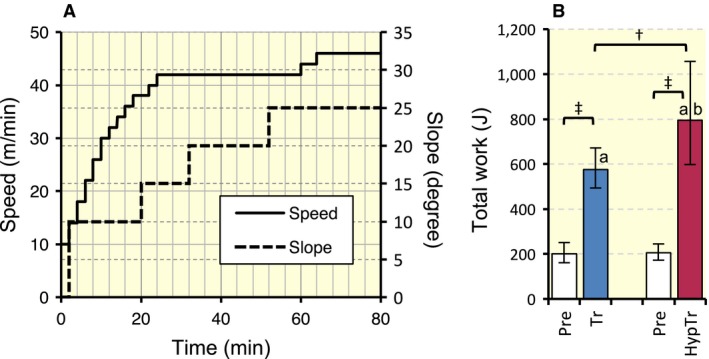
(A) Graded ramp treadmill running protocol. (B) Total work capacity after 4 weeks of exercise training with and without hyperbaric exposure. Values are represented as means ± SD. ^†^ and ^‡^, Significantly different at *P* < 0.01 and *P* < 0.001, respectively, using the Student's paired or unpaired *t*‐test. a and b, The 95% confidential interval did not contain the parameter value specified in the null hypothesis between the Pre (pre‐training values) and Tr groups, respectively.

Following the acclimation period, mice in the training group were subjected to a maximal exercise capacity test with a graded ramp running protocol using the controlled treadmill, as shown in Figure 1A. Total work (joules) was calculated by the product of body weight (kg), speed (m sec^−1^), time (sec), slope (%), and 9.8 (m sec^−2^). After the test, mice in the training group were divided into an exercise‐trained group (Tr, *n* = 10) or intermittent hyperbaric exposure with exercise‐trained group (HypTr, *n* = 10) to match the mean and standard deviation (SD) values of pre‐training (Pre) results of total work (Fig. 1B).

The Hyp and HypTr groups was subjected to hyperbaric exposure as described in Experiment 1 for 1 h, once per day (from 5 am to 6 am), 6 days per week for 4 weeks. For the HypTr group, hyperbaric exposure and exercise training were performed separately.

Mice in the training groups were subjected to endurance exercise training for 4 weeks, 6 days per week using a rodent treadmill (KN‐73, Natsume Co., Tokyo, Japan). Mice ran for 50 min at 18 m min^−1^ with a 10 deg incline on the first day of training. On the fourth day, the duration was increased to 60 min. Running speed was gradually increased to 24 m min^−1^ throughout the training period. Daily exercise training was performed from 5 am to 6 am for the Tr group and from 6:30 am to 7:30 am for the HypTr group. Following the training regimen, trained mice ran on the treadmill for 20 min at 18 m min^−1^ with a 5 deg incline. The maximal exercise capacity test was performed 24 h after the last run and 48 h after the last hyperbaric exposure, as described above.

Thirty hours after the exercise capacity test, mice were anesthetized and muscles were excised as described above. The remaining muscles, that is the right side, were excised and placed in embedding medium, O.C.T. compound (Miles Inc., Elkhart, IN), and then rapidly frozen in isopentane cooled to its melting point (−160°C) with liquid nitrogen. All tissue samples were stored at −80°C until later analyses.

#### Histological analyses

**Figure 2:**
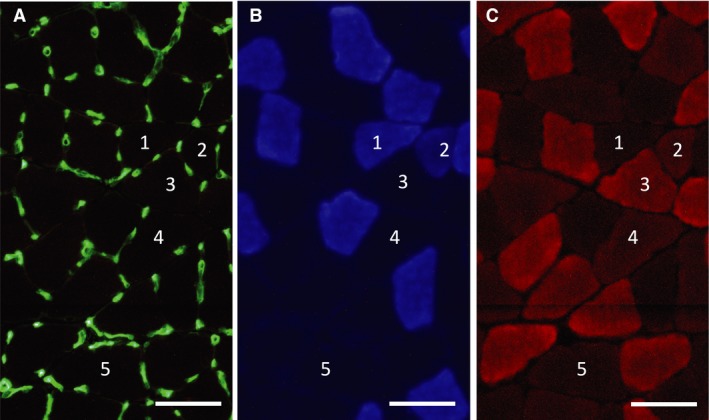
Representative immunofluorescent images of capillary (A) and type I (B) and type IIA muscle fibers. 1: Type I fiber; 2: type I + IIA fiber; 3: type IIA fiber; 4: type IIAX fiber; 5: type IIX fiber. Horizontal bars represent 50 *μ*m.

Ten‐micrometer‐thick serial cross‐sections were obtained using a cryotome (CM‐1500; Leica Japan Inc., Tokyo, Japan) at −20°C from the mid‐belly portion of calf muscles. These sections were air‐dried, fixed with 100% methanol for 15 min at 4°C, and then washed in 0.1 mol L^−1^ phosphate‐buffered saline with 0.05% Triton X‐100 (PBS‐T). Sections were then blocked with 10% goat normal serum at room temperature for 1 h. To examine capillary profiles and muscle fiber phenotypes, sections were incubated at 4°C overnight with a mixture of fluorescein‐labelled Griffonia simplicifolia lectin (GSL I) (FL 1101 (1:300), Vector Laboratories Inc., CA), an anti‐type I myosin heavy chain (MHC) antibody (BA‐F8, mouse IgG2b, 1:50), and anti‐type IIA MHC antibody (SC‐71, mouse IgG1, 1:100) diluted with PBS‐T containing 5% goat normal serum. After washing three times with PBS‐T, sections were reacted with a secondary antibody mixture containing Alexa Fluor 350‐labelled anti‐mouse IgG2b (1:100, A21140, blue) and Alexa Fluor 555‐labelled anti‐mouse IgG1 (1:100, A21127, red) diluted with PBS‐T at room temperature for 1 h. Sections were washed three times with PBS‐T, air‐dried, and coverslipped with Fluoromount/Plus (K048, Diagnostic BioSystems Co., CA). Primary and secondary antibodies were purchased from the Developmental Studies Hybridoma Bank (University of Iowa) and Thermo Fisher Scientific Inc. (Tokyo, Japan), respectively. Fluorescent images of the incubated sections were observed using a microscope (Axio Observer, Carl Zeiss Japan, Tokyo, Japan). Three individual images using blue (Filter Set 49, excitation 365/emission 445), red (Filter Set 43, 545/605), or green (Filter Set 38, 470/525) filters were taken at one microscopic field and stored on a computer disk. Muscle fiber phenotypes were classified as type I (blue), type I + IIA (faint blue and faint red), type IIA (red), type IIAX (faint red), and type IIX (unstained). Non‐overlapping microscopic fields were selected at random from each muscle sample. The observer was blind to the source (groups) of each slide during the measurements. Fluorescent images were obtained from SOL, PL, Gr, and Gw. Representative immunofluorescent images were shown in Figure 2. It was confirmed that negative control without primary antibodies showed no fluorescent signal.

To assess the expression of PGC‐1*α*, 10‐*μ*m‐thick cross‐sections were air‐dried, fixed with 3.8% formaldehyde in PBS for 15 min, and incubated at 4°C overnight with an Alexa Fluor 488‐labelled anti‐PGC‐1*α* antibody (1:500, Novus Biologicals, CO) diluted with PBS‐T. After washing three times with PBS‐T, sections were reacted with 4′, 6‐diamidino‐2‐phenylindole dihydrochloride (DAPI, D9542, Sigma‐Aldrich, MO) to stain the nucleus blue in fluorescent color. Slides were then rinsed with distilled water and mounted as described above. Two individual images were taken using the respective filter sets (Filter Set 49 or Filter Set 38) at one microscopic field with a constant exposure time for each image and stored on a computer disk. To assess the expression of PGC‐1*α* in the nucleus, each RGB image was split into a green (PGC‐1*α*) or blue (nucleus) channel and converted to an 8‐bit greyscale image using ImageJ software. The colocalized area of two images was obtained with a constant threshold value for each image. All fluorescent images were obtained within 24 h of staining.

#### Biochemical analyses of enzyme activity

Frozen tissue powder was obtained using a frozen sample crusher (SK mill, Tokken Inc., Chiba, Japan) and homogenised with ice‐cold medium (10 mmol L^−1^ HEPES buffer, pH 7.4; 0.1% Triton X‐100; 11.5% (w/v) sucrose; and 5% (v/v) protease inhibitor cocktail (P2714, Sigma‐Aldrich). After centrifugation at 1500*g* at 4°C for 10 min, the supernatant was used in enzyme activity analyses. The activities of 3‐hydroxyacyl‐CoA‐dehydrogenase (HAD) and lactate dehydrogenase (LDH) were assayed according to the method of Bass et al. (1969). The activities of citrate synthase (CS) and phosphofructokinase (PFK) were assayed according to the method of Srere (1969) and Passonneau and Lowry (1993), respectively. Total carnitine palmitoyl transferase (CPT) activity was assayed according to the method of (Abel 2004). All measurements were conducted at 25°C with a spectrophotometer (U‐2001, Hitachi Co., Tokyo, Japan) and enzyme activities were obtained as *μ*mol h^−1^ g protein^−1^. Total protein concentrations were measured using PRO‐MEASURE protein measurement solution (iNtRON Biotechnology Inc., Gyeonggi‐do, Korea).

#### Western blot analyses

The tissue homogenates described above were used for Western blot analyses. A sample was fractionated by SDS/PAGE on 7.5% or 12% (w/v) polyacrylamide gels (TGX StainFree FastCast gel, Bio‐Rad Inc., CA) and electrophoretically transferred to a polyvinylidene fluoride membrane. The bands in each lane on the membrane were detected with a UV imager (ChemiDoc, Bio‐Rad). The blots were blocked with 3% (w/v) bovine serum albumin, 1% (w/v) polyvinylpyrrolidone, and 0.3% (v/v) Tween‐20 in PBS for 1 h, and then exposed to a specific primary antibody (Santa Cruz Biotechnology Inc., CA) against HSP70 (1:500, sc‐1060) or Tfam (1:500, sc‐166965) diluted in PBS with 0.05% Tween‐20 for 1 h. After the blots had been incubated with a HRP‐labelled secondary antibody (Santa Cruz), they were reacted with Clarity Western ECL substrate (Bio Rad) and the required proteins were detected with an imager (ChemiDoc). The densities of the bands were normalised to the densities of all protein bands in each lane on the membrane (Gilda and Gomes 2013; Vigelsø et al. 2014). The densities of the bands were quantified using Image Lab software (Bio Rad) and normalised to the same sample that was run on every gel and transferred to every membrane.

### Statistical analyses

All values are transformed using base‐10 logs. In experiment 1, differences in the values between the Sed group and each time point were assessed using Dunnett's multiple comparison procedure. In experiment 2, a two‐way ANOVA was used to assess the interaction of exercise training and hyperbaric exposure. Differences among the four groups were examined using the Tukey‐Kramer multiple comparisons test. Differences in maximal exercise performance before and after training with or without hyperbaric exposure were analyzed using the Student's paired *t*‐test. Mean, standard deviation (SD), and 95% confidential interval (CI) values were expressed after back‐transformation.

## Results

### Experiment 1. Time‐course responses to acute hyperbaric exposure

**Figure 3:**
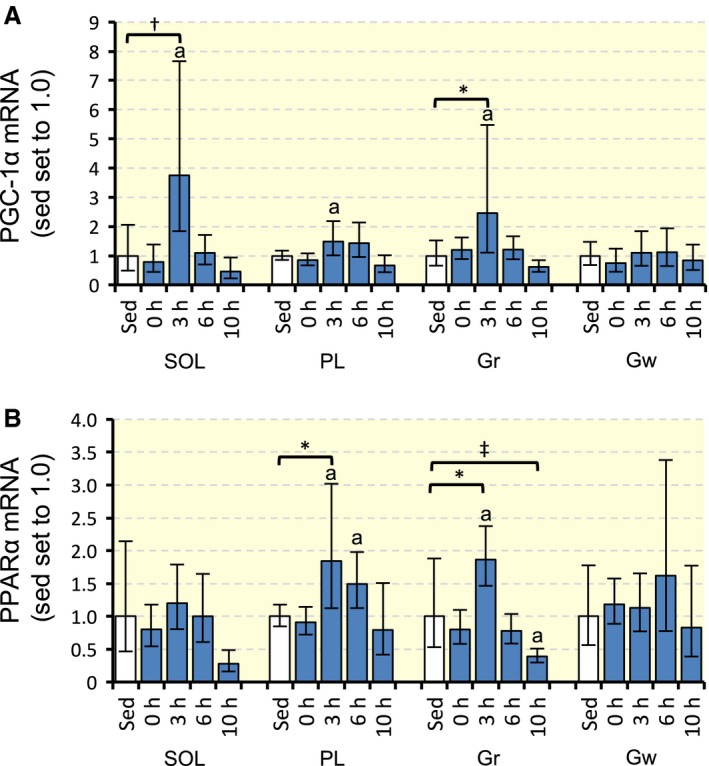
Expression of PGC‐1*α* (A) and PPAR *α* (B) mRNA after acute hyperbaric exposure. Values are represented as means ± SD. *, ^†^, and ^‡^, Significantly different at *P* < 0.05, *P* < 0.01, and *P* < 0.001, respectively, using Dunnett's multiple comparison. a, The 95% confidential interval did not contain the parameter value specified in the null hypothesis between the Sed group and each time point.

The expression of PGC‐1*α* mRNA was significantly stronger in SOL (7.2‐fold, *P* < 0.01) and Gr (5.1‐fold, *P* < 0.05) 3 h after hyperbaric exposure than in Sed (Fig. 3A). In PL, marked increases in PGC‐1*α* mRNA levels were observed 3 (2.1‐fold) and 6 h (2.1‐fold) after hyperbaric exposure.

PPAR*α* mRNA levels significantly increased in PL (2.9‐fold, *P* < 0.05) 3 h after hyperbaric exposure (Fig. 3B). In Gr, the expression of PPAR*α* mRNA increased 3 h after (2.3‐fold, *P* < 0.05) and decreased 10 h (0.50‐fold, *P* < 0.001) after hyperbaric exposure.

### Experiment 2. Chronic response to exercise training with hyperbaric exposure

#### Muscle and heart masses

SOL muscle weight significantly increased after training with hyperbaric exposure (Table 1, *P* < 0.05). The relative weight of SOL was significantly greater in the Tr and HypTr groups than in the Sed group. Whole heart and left ventricle weights were significantly greater in the HypTr group than in the three other groups (*P* < 0.05). The relative weights of the whole heart and left ventricle were significantly greater in the Tr and HypTr groups than in the Sed group (*P* < 0.05).

**Table 1:** Body and organ masses

	Sed (*n* = 9)	Hyp (*n* = 8)	Tr (*n* = 10)	HypTr (*n* = 10)	Two‐way ANOVA		
					E	H	I
Body mass (g)	39.4 ± 1.4/1.3	40.4 ± 1.1/1.1^1^	37.1 ± 2.2/2.1^8^	39.8 ± 2.7/2.5[Link], [Link]	4	4	ns
Organ mass (mg)							
Gastrocnemius	178.3 ± 6.6/6.4	172.4 ± 10.9/10.2	175.7 ± 9.6/9.1^2^	182.2 ± 11.9/11.1^1,2,3^	ns	ns	ns
Soleus	9.5 ± 0.47/0.45	9.7 ± 0.64/0.60^1^	9.8 ± 0.83/0.77^1^	10.7 ± 1.6/1.4[Link], [Link]	4	ns	ns
Plantaris	22.0 ± 1.9/1.7	20.4 ± 3.1/2.7	21.8 ± 3.9/3.3^2^	22.4 ± 2.5/2.2^2,3^	ns	ns	ns
Whole heart	159.3 ± 8.6/8.1	165.4 ± 5.8/5.6^1^	163.2 ± 14.0/12.9^1^	183.1 ± 18.6/16.9[Link], [Link]	4	5	ns
Left ventricle	111.9 ± 5.9/5.6	111.7 ± 3.2/3.2^1^	120.2 ± 10.8/9.9^1,2^	133.0 ± 12.7/11.6[Link], [Link]	6	4	ns
Organ mass‐to‐body mass ratio (mg g^−1^)							
Gastrocnemius	4.53 ± 0.25/0.24	4.27 ± 0.24/0.23^7^	4.73 ± 0.12/0.11[Link], [Link]	4.57 ± 0.14/0.13[Link], [Link]	6	5	ns
Soleus	0.24 ± 0.02/0.01	0.24 ± 0.02/0.02	0.26 ± 0.02/0.02[Link], [Link]	0.27 ± 0.03/0.02[Link], [Link]	6	ns	ns
Plantaris	0.56 ± 0.06/0.05	0.50 ± 0.07/0.06	0.59 ± 0.09/0.08^1,2^	0.56 ± 0.06/0.06[Link], [Link]	ns	ns	ns
Whole heart	4.05 ± 0.19/0.19	4.09 ± 0.16/0.16^1^	4.38 ± 0.30/0.19[Link], [Link]	4.59 ± 0.33/0.31[Link], [Link]	6	ns	ns
Left ventricle	2.83 ± 0.14/0.13	2.91 ± 0.07/0.07^1^	3.22 ± 0.20/0.19[Link], [Link]	3.34 ± 0.24/0.23[Link], [Link]	6	ns	ns

Values are represented as means ± SD (upper/lower).

^1,2,3^The 95% confidential interval did not contain the parameter value specified in the null hypothesis between the Sed, Hyp, or Tr groups, respectively.

^4,5,6^Effects of exercise (E), hyperbaric exposure (H), and their interaction (I) were significantly different at *P* < 0.05, *P* < 0.01, and *P* < 0.001, respectively, using a two‐way ANOVA.

^7,8,9^Significantly different from the Sed, Hyp, and Tr groups, respectively, at *P* *<* 0.05 using the Tukey‐Kramer multiple comparisons test.

#### Maximal exercise capacity

Maximal exercise capacity expressed in total work (joules) significantly increased after training with (3.9‐fold, *P* < 0.001) and without (2.9‐fold, *P* < 0.001) hyperbaric exposure (Fig. 1B). Moreover, total work values were significantly greater in the HypTr group than in the Tr group (1.4‐fold, *P* < 0.01). Thus, hyperbaric exposure had additive effects on exercise‐induced improvements in endurance exercise capacity.

#### Fiber type composition

**Figure 4:**
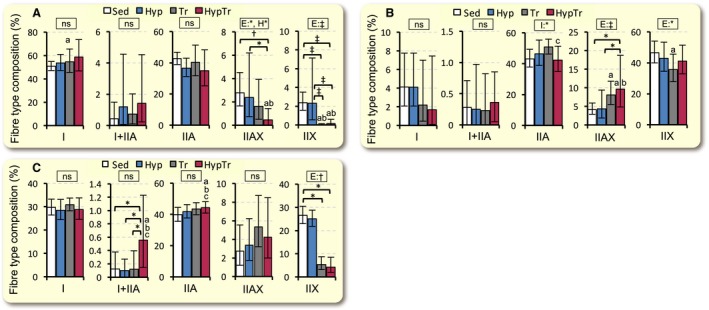
Fiber type composition values in SOL (A), PL (B), and Gr (C) after 4 weeks of exercise training with and without hyperbaric exposure. Values are represented as means ± SD. *, ^†^, and ^‡^, Significantly different at *P* < 0.05, *P* < 0.01, and *P* < 0.001, respectively, using a two‐way ANOVA (represented in the rectangular box, E, exercise; H, hyperbaric exposure; I, interaction; ns, not significant) or the Tukey‐Kramer multiple comparisons test. a, b, and c, The 95% confidential interval did not contain the parameter value specified in the null hypothesis between the Sed, Hyp, or Tr groups, respectively.

The proportion of type I + IIA fibers in Gr was significantly greater (4.5–5.6‐fold) in the HypTr group than in the three other groups (*P* < 0.05, Fig. 4C). In the training groups with and without intermittent hypoxia, the proportion of type IIX fibers was greater than that in the Sed group in SOL (*P* < 0.001, Fig. 4A) and Gr (*P* < 0.05, Fig. 4C). In SOL, the proportion of type IIAX fibers was markedly greater in the HypTr group than in the Sed group (*P* < 0.05). In PL, the proportion of type IIAX fibers was significantly greater in the HypTr group (2.3‐fold and 2.2‐fold, respectively) than in the Sed and Hyp groups (*P* < 0.05). Gw was composed of type IIX fibers only (data not shown).

#### Capillarization

Capillary‐to‐fiber (C:F) ratio values in SOL were significantly greater in the Tr group than in the Sed group (*P* < 0.05, Table 2). In the PL, the C:F values was significantly greater in the Tr (*P* < 0.05) and HypTr (*P* < 0.01) groups than in the Sed group. Capillary density value was greater in the Tr group only than in the Sed group in PL (*P* < 0.05, Table 2). Thus, daily hyperbaric exposure did not facilitate exercise‐induced capillary growth in the hindlimb muscles.

**Table 2:** Capillary‐to‐fiber ratio and capillary density values

	Sed	Hyp	Tr	HypTr	Two‐way ANOVA		
					E	H	I
Capillary‐to‐fiber ratio							
SOL	1.72 ± 0.08/0.07	1.81 ± 0.14/0.13	1.93 ± 0.15/0.14[Link], [Link]	1.89 ± 0.26/0.23^1^	5	ns	ns
PL	1.60 ± 0.12/0.11	1.64 ± 0.17/0.16	1.80 ± 0.14/0.13[Link], [Link]	1.78 ± 0.09/0.08[Link], [Link]	6	ns	ns
Gr	1.89 ± 0.13/0.12	1.86 ± 0.10/0.09	2.01 ± 0.15/0.14^2^	2.05 ± 0.22/0.20^2^	4	ns	ns
Gw	1.02 ± 0.15/0.13	1.01 ± 0.14/0.13	0.98 ± 0.17/0.14	1.09 ± 0.26/0.21	ns	ns	ns
Capillary density (no. mm^−2^)							
SOL	984.8 ± 206.9/171.0	998.9 ± 182.5/154.3	1162.9 ± 193.3/165.7^1,2^	1187.0 ± 226.6/190.3^1,2^	5	ns	ns
PL	910.3 ± 166.5/140.7	869.0 ± 193.9/158.6	1122.6 ± 85.0/79.0[Link], [Link]	975.0 ± 134.2/118.0^2^	4	4	ns
Gr	1086.0 ± 180.9/155.1	972.3 ± 163.0/139.6	1299.5 ± 395.3/303.1[Link], [Link]	1306.8 ± 304.9/247.2[Link], [Link]	5	ns	ns
Gw	468.9 ± 74.0/63.9	421.9 ± 90.3/74.4	447.0 ± 66.0/57.5	468.3 ± 89.3/75.0	ns	ns	ns

Values are represented as means ± SD (upper/lower).

^1,2^The 95% confidential interval did not contain the parameter value specified in the null hypothesis between the Sed or Hyp groups, respectively.

^3,4,5^Effects of exercise (E), hyperbaric exposure (H), and their interaction (I) were significantly different at *P* < 0.05, *P* < 0.01, and *P* < 0.001, respectively, using a two‐way ANOVA.

^6,7^Significantly different from the Sed and Hyp groups, respectively, at *P* < 0.05 using the Tukey‐Kramer multiple comparisons test.

#### Metabolic enzyme activity

CS activity values were significantly greater in the HypTr group than in the Sed group in all muscle portions examined (Table 3), whereas these values were greater in the Tr group than in the Sed group in PL (*P* < 0.01) and Gr (*P* < 0.001). Moreover, CS activity values were significantly greater in PL (1.3‐fold, *P* < 0.001) and Gw (1.3‐fold, *P* < 0.01) in the HypTr group than in the Tr group. In CS activity values in Gw, the interaction effect of exercise and hyperbaric exposure was significant (two‐way ANOVA, *P* = 0.015).

**Table 3:** Enzyme activity values

		Sed	Hyp	Tr	HypTr	Two‐way ANOVA		
						E	H	I
CS	SOL	8.4 ± 0.88/0.80	7.8 ± 0.87/0.78	9.9 ± 1.7/1.5[Link], [Link]	9.9 ± 1.7/1.5[Link], [Link]	6	ns	ns
	PL	8.6 ± 0.66/0.61	7.6 ± 0.64/0.59[Link], [Link]	10.0 ± 1.0/0.93[Link], [Link]	13.1 ± 1.1/0.99[Link], [Link]	6	ns	6
	Gr	11.0 ± 0.87/0.81	11.2 ± 0.90/0.83	14.8 ± 1.5/1.4[Link], [Link]	15.3 ± 1.7/1.5[Link], [Link]	6	ns	ns
	Gw	5.0 ± 0.84/0.72	4.8 ± 0.79/0.68	5.0 ± 0.85/0.73	6.4 ± 1.1/0.92[Link], [Link]	4	4	ns
HAD	SOL	1.5 ± 0.30/0.25	1.4 ± 0.41/0.32	1.5 ± 0.63/0.44	2.0 ± 0.43/0.36[Link], [Link]	5	ns	ns
	PL	0.97 ± 0.07/0.07	0.98 ± 0.16/0.14	1.2 ± 0.19/0.16[Link], [Link]	1.6 ± 0.38/0.31[Link], [Link]	6	ns	4
	Gr	1.3 ± 0.39/0.30	1.5 ± 0.23/0.20	2.2 ± 0.25/0.22[Link], [Link]	2.2 ± 0.30/0.26[Link], [Link]	6	ns	ns
	Gw	0.44 ± 0.13/0.10	0.42 ± 0.11/0.09	0.46 ± 0.11/0.09	0.48 ± 0.14/0.11	ns	ns	ns
Total CPT	SOL	0.46 ± 0.11/0.09	0.44 ± 0.09/0.07	0.44 ± 0.15/0.11	0.45 ± 0.04/0.04	ns	ns	ns
	PL	0.38 ± 0.05/0.05	0.38 ± 0.09/0.07	0.43 ± 0.06/0.06^1,2^	0.48 ± 0.03/0.03[Link], [Link]	6	ns	ns
	Gr	0.41 ± 0.09/0.08	0.30 ± 0.08/0.06[Link], [Link]	0.44 ± 0.04/0.04[Link], [Link]	0.45 ± 0.04/0.03[Link], [Link]	6	ns	ns
	Gw	0.13 ± 0.07/0.05	0.12 ± 0.07/0.04	0.18 ± 0.07/0.05	0.15 ± 0.06/0.04	ns	ns	ns
LDH	SOL	28.6 ± 3.3/2.9	27.3 ± 6.3/5.1	22.8 ± 2.6/2.3[Link], [Link]	23.5 ± 3.9/3.4[Link], [Link]	6	ns	ns
	PL	90.4 ± 19.4/15.9	84.6 ± 14.2/12.2	77.6 ± 18.1/14.7	84.0 ± 19.2/15.7	ns	ns	ns
	Gr	69.4 ± 13.9/11.6	82.0 ± 3.3/3.2^1^	97.5 ± 13.2/11.6[Link], [Link]	98.8 ± 16.2/14.0[Link], [Link]	6	ns	ns
	Gw	114.1 ± 6.7/6.3	112.5 ± 7.8/7.3	107.2 ± 3.8/3.7^1^	105.8 ± 9.8/8.9^1^	4	ns	ns
PFK	SOL	0.42 ± 0.39/0.20	0.74 ± 0.91/0.41	0.57 ± 0.56/0.28	0.58 ± 0.92/0.36	ns	ns	ns
	PL	6.6 ± 2.9/2.0	7.6 ± 2.2/1.7	5.8 ± 2.7/1.8^2^	8.7 ± 2.9/2.2[Link], [Link]	ns	ns	ns
	Gr	7.8 ± 4.5/2.8	4.2 ± 2.8/1.7[Link], [Link]	9.7 ± 1.0/0.88[Link], [Link]	6.0 ± 3.9/2.4^2,3^	ns	5	ns
	Gw	7.0 ± 1.3/1.1	6.5 ± 4.9/2.8	9.3 ± 3.1/2.3^1,2^	12.6 ± 2.6/2.2[Link], [Link]	5	ns	ns

Values are represented as means ± SD (upper/lower). CS, citrate synthase; HAD, 3‐hydroxyacyl‐CoA‐dehydrogenase; CPT, carnitine palmitoyl transferase; LDH, lactate dehydrogenase; PFK, phosphofructokinase.

^1,2,3^The 95% confidential interval did not contain the parameter value specified in the null hypothesis between the Sed, Hyp, or Tr groups, respectively.

^4,5,6^Effects of exercise (E), hyperbaric exposure (H), and their interaction (I) were significantly different at *P* < 0.05, *P* < 0.01, and *P* < 0.001, respectively, using a two‐way ANOVA.

^7,8,9^Significantly different from the Sed, Hyp, and Tr groups, respectively, at *P* < 0.05 using the Tukey‐Kramer multiple comparisons test.

In PL, HAD activity values were significantly greater in the Tr and HypTr groups than in the Sed group, and were markedly greater in the HypTr group than in the Tr group (1.4‐fold, *P* < 0.001). In HAD activity values in PL, the interaction effect in the two‐way ANOVA was significant (*P* = 0.018). In SOL, HAD activity values were markedly greater in the HypTr group than in the Tr group (1.4‐fold, 95% CI: 1.2–1.6, *P* = 0.080).

Total CPT activity values were only significantly greater in PL in the HypTr group than in the Sed group (*P* < 0.001).

In PL, PFK activity values were significantly greater in the HypTr group than in the Tr group (1.5‐fold, *P* < 0.05). In Gw, PFK activity values were markedly greater in the HypTr group than in the Sed (1.8‐fold, *P* < 0.01) and Tr (1.4‐fold, 95% CI: 1.2–1.5, *P* = 0.18) groups.

LDH activity values were significantly greater in the Tr and HypTr groups than in the Sed group in Gr (*P* < 0.001), but these values were lower in SOL (*P* < 0.05). Thus, chronic intermittent hyperbaric exposure increased mitochondrial oxidative enzyme activities as well as glycolytic enzyme activities in hindlimb muscles.

#### PGC‐1α expression

**Figure 5:**
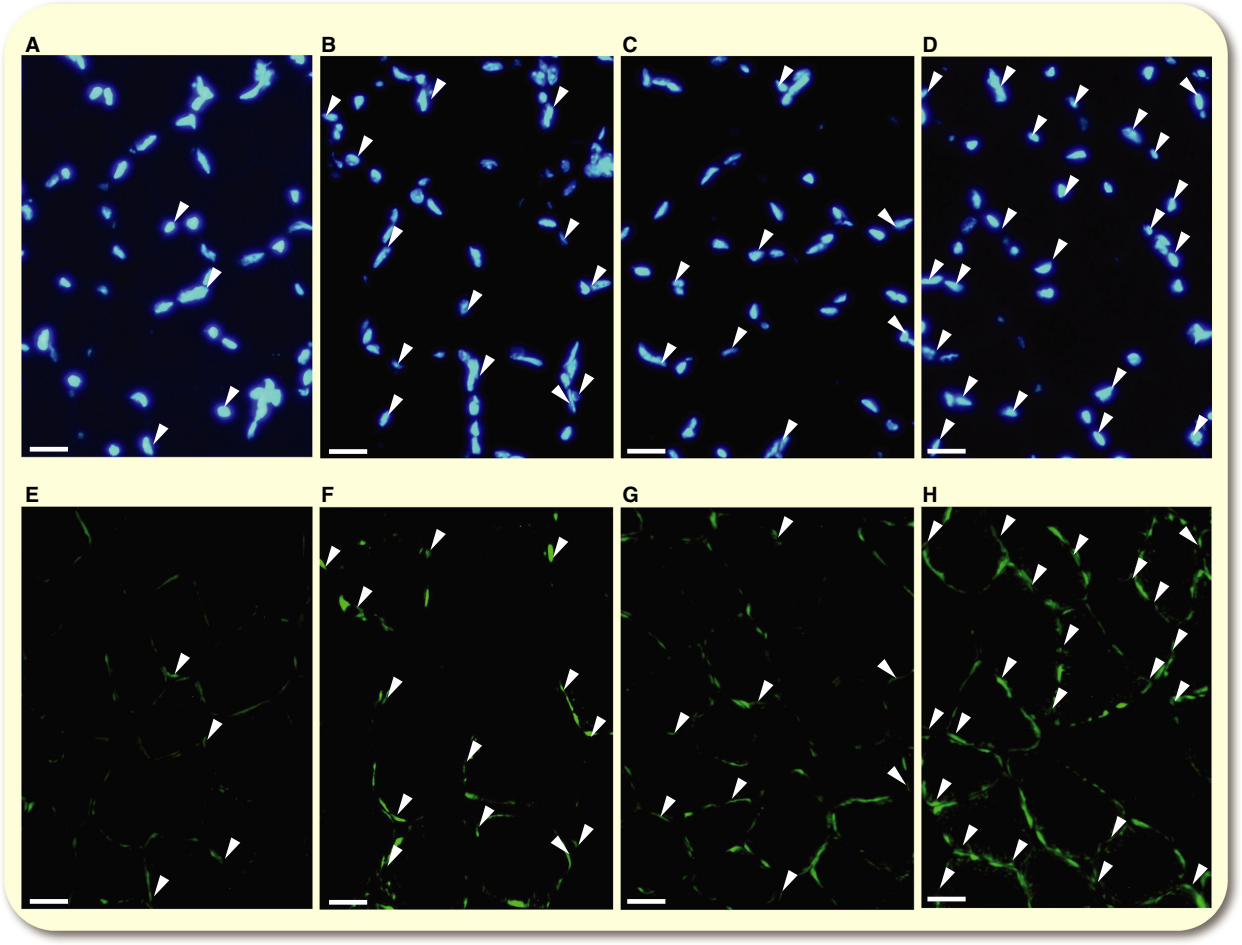
Representative immunofluorescent images of the nucleus (A–D) and PGC‐1*α* (E‐F) in Gr in Sed (A and E), Hyp (B and F), Tr (C and G), and HypTr (D and H) groups. Arrow heads represent the colocalized area for PGC‐1 *α* and the nucleus. Horizontal bars represent 20 *μ*m.

**Figure 6:**
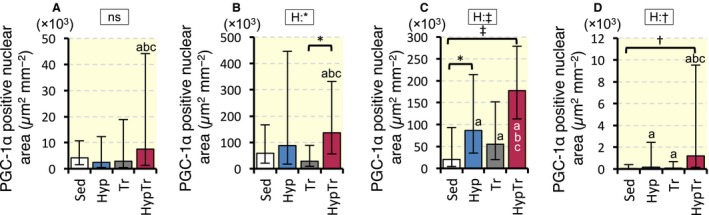
Colocalized area values for PGC‐1 *α* and the nucleus in SOL (A), PL (B), Gr (C), and Gw (D). Values are represented as means ± SD. *, †, and ‡, significantly different at *P* < 0.05, *P* < 0.01, and *P* < 0.001, respectively, using a two‐way ANOVA (represented in the rectangular box, E, exercise; H, hyperbaric exposure; I, interaction; ns, not significant) or the Tukey‐Kramer multiple comparisons test. a, b, and c, The 95% confidential interval did not contain the parameter value specified in the null hypothesis between the Sed, Hyp, or Tr groups, respectively.

Representative fluorescent images in Gr are shown in Figure 5. The PGC‐1*α*‐positive nuclear area was significantly larger in the HypTr group than in the Sed group in Gr (8.9‐fold, *P* < 0.001, Fig. 6C) and Gw (37‐fold, *P* < 0.01, Fig. 6D). PGC‐1*α* expression in the nucleus was markedly greater in the HypTr group than in the Tr group in PL (4.8‐fold, 95% CI: 4.0–5.8, *P* = 0.026, Fig. 6B), Gr (3.2‐fold, 95% CI: 2.9–3.5, *P* = 0.077, Fig. 6C), and Gw (15‐fold, 95% CI: 9.7–22.2, *P* = 0.066, Fig. 6D). Thus, chronic intermittent hyperbaric exposure facilitated the expression of the PGC‐1*α* protein in the nucleus in the hindlimb muscles.

#### Protein expression

**Figure 7:**
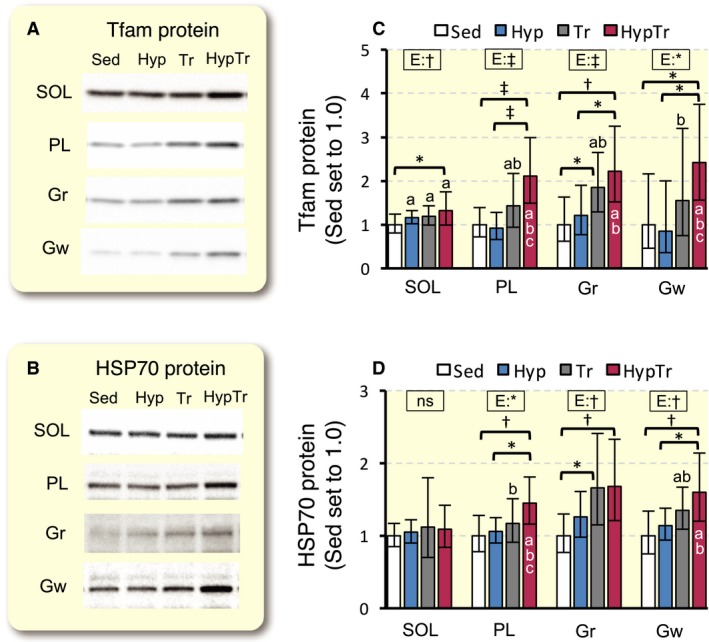
Expression of Tfam (A and B) and HSP70 (C and D) proteins after 4 weeks of exercise training with and without hyperbaric exposure. Values are represented as means ± SD. *, †, and ‡, significantly different at *P* < 0.05, *P* < 0.01, and *P* < 0.001, respectively, using a two‐way ANOVA (represented in the rectangular box, E, exercise; H, hyperbaric exposure; I, interaction; ns, not significant) or the Tukey‐Kramer multiple comparisons test. a, b, and c, The 95% confidential interval did not contain the parameter value specified in the null hypothesis between the Sed, Hyp, or Tr groups, respectively.

The representative expression of the Tfam protein was shown in Figure 7A. Tfam protein expression was significantly greater in the HypTr group than in the Sed group in SOL (1.3‐fold, *P* < 0.05), PL (1.5‐fold, *P* < 0.001), and Gw (1.6‐fold, *P* < 0.05, Fig. 7C). The expression of the Tfam protein was markedly greater in the HypTr group than in the Tr group in PL (1.5‐fold, 95% CI: 1.2–1.9, *P* = 0.097) and Gw (1.6‐fold, 95% CI: 1.2–2.1, *P* = 0.50). In Gr, Tfam expression was significantly greater in the Tr and HypTr groups than in the Sed group (2.9‐fold and 1.9‐fold, respectively).

The representative expression of the HSP70 protein was shown in Figure 7B. HSP70 expression was significantly greater in the HypTr group, but not in the training group, than in the Sed group in PL (1.5‐fold, *P* < 0.01) and Gw (1.2‐fold, *P* < 0.01). The expression of the HSP70 protein was markedly greater in the Tr (1.7‐fold, *P* < 0.05) and HypTr (1.7‐fold, *P* < 0.01) groups than in the Sed group in Gr.

## Discussion

The principal result of this study was that intermittent hyperbaric exposure promoted endurance exercise performance by facilitating oxidative and glycolytic capacities and the expression of proteins involved in mitochondrial biogenesis in hindlimb muscles.

### Time‐course responses to acute hyperbaric exposure

In this study, PGC‐1*α* mRNA levels in SOL and Gr markedly increased 3 h after acute hyperbaric exposure (Fig. 3A). This result is partly supported by a previous study showing that acute hyperbaric exposure (1.25 ATA, 20.9% O_2_) for 1 h up‐regulated PGC‐1*α* mRNA levels 1 h after exposure in SOL and the extensor digitorum longus in rats (Fujita et al. 2014) PGC‐1*α* was shown to stimulate energy production by activating downstream genes involved in fatty acid and glucose metabolism in skeletal muscles (Benton et al. 2008, 2010).

This study is the first to demonstrate that PPAR*α* mRNA levels increased in PL and Gr 3 h after acute hyperbaric exposure (Fig. 3B). PGC‐1*α* was shown to act as a coactivator of PPAR*α* and up‐regulated PPAR*α* target genes involved in the mitochondrial fatty acid oxidation pathway (Benton et al. 2010)

Acute exercise up‐regulated PGC‐1*α* mRNA levels in Gr after 3‐6 h and in Gw after 0‐10 h (Suzuki 2016). Thus, chronic exercise training with hyperbaric exposure may have additive effects on mitochondrial adaptation to exercise, thereby promoting exercise performance.

### Chronic response to exercise training with intermittent hyperbaric exposure

This study showed that expression of the PGC‐1*α* protein in the nucleus markedly increased after exercise training with intermittent hyperbaric exposure, but did not after training alone (Fig. 6). Thus, intermittent hyperbaric exposure with exercise facilitated the accumulation of the PGC‐1*α* protein in skeletal muscle. The activation of p38 mitogen‐activated protein kinase (MAPK) was shown to stabilize PGC‐1*α* in vitro (Puigserver et al. 2001) 5′ AMP‐activated protein kinase (AMPK) was reported to facilitate translocation of the PGC‐1*α* protein to the nucleus and mitochondria (Safdar et al. 2011). The phosphorylation of p38MAPK was markedly facilitated during 1‐4 h of hyperbaric exposure (2.5 ATA, 98% O_2_ + 2% CO_2_) in cultured vascular smooth muscle cells (Shyu et al. 2004). However, the up‐regulation of these kinases has not been reported after hyperbaric exposure, similar to that used in this study. Further investigations are needed to elucidate the mechanisms underlying the up‐regulation of PGC‐1*α* in the nucleus by chronic intermittent hyperbaric exposure with exercise.

The expression of Tfam was mainly regulated by PGC‐1*α* and a complex of these two proteins promoted mitochondrial biogenesis by the transcription of mitochondrial genome‐encoded genes and mtDNA replication (Wu et al. 1999) In this study, exercise training with hyperbaric exposure markedly increased Tfam protein expression in all muscle portions examined, whereas training alone only enhanced it in Gr (Fig. 7A and C). Swimming exercise training for 7 days markedly enhanced Tfam protein expression and reduced miR‐494 expression, which post‐transcriptionally inhibits Tfam expression, when muscle samples were collected 2 h after the last training bout in mice (Yamamoto et al. 2012). In humans, 4 weeks of exercise training with restricted blood flow failed to facilitate exercise‐induced Tfam protein expression when muscle biopsy samples were collected 24 h after the last exercise bout (Bengtsson et al. 2001). Therefore, expression levels of the Tfam protein may decrease with time after an exercise bout. In this study, an apparent increase in Tfam protein expression was observed 30 h after the last exercise bout in the HypTr group only. Thus, exercise training with hyperbaric exposure may facilitate mitochondrial biogenesis via the up‐regulation of PGC‐1*α* and Tfam.

In metabolic syndrome (SHR/NDmcr‐cp) rats, hyperbaric exposure (1.25 ATA with 36% O_2_) 3 h daily for 16 weeks restored succinate dehydrogenase staining intensity in SOL to a normal control level (Takemura and Ishihara 2017). However, this study is the first to measure metabolic enzyme activities after exercise training with hyperbaric exposure. In PL, CS, HAD, and PFK activity levels were significantly greater in the HypTr group than in the Tr group (Table 3). Furthermore, total CPT activity markedly increased after training with hyperbaric exposure, but not after training alone. Thus, the capacities of fatty acid and glucose metabolism were facilitated after exercise training with hyperbaric exposure in muscle that was mainly composed of type IIA and IIX fibers. In Gw, CS activity levels were significantly greater in the HypTr group than in the Tr group. Moreover, PFK activity levels markedly increased after training with hyperbaric exposure, but not after training alone. Thus, the capacity for glycolysis and oxidative phosphorylation was strongly promoted by training with hyperbaric exposure in muscle composed of type IIX fibers only. This study appears to be the first to report these adaptive changes in fast‐twitch muscle after endurance exercise training. These adaptive changes in enzyme activity were induced by the up‐regulation of PGC‐1*α* and Tfam proteins, and contributed to increases in endurance exercise capacity.

Endurance training for 6 weeks did not change the relative proportion of hybrid fibers using a single fiber electrophoresis method (Glaser et al. 2010). This notion is consistent with the results obtained from the Tr group in this study (Fig. 4). After training with hyperbaric exposure, the composition of hybrid type I + IIA fibers significantly increased in Gr (Fig. 4C), while that of hybrid type IIAX fibers markedly increased in PL (Fig. 4B). In contrast, the relative composition of type IIAX fibers significantly decreased after training with hyperbaric exposure in SOL (Fig. 4A). Thus, exercise training with intermittent hyperbaric exposure may alter the expression of hybrid fibers. Hybrid fibers are considered to be transitional between one pure type and another (Pette and Staron 2001). However, the physiological meaning of these intermediate phenotypes currently remains unclear.

HSP70 protein expression levels markedly increased after exercise training with intermittent hyperbaric exposure, but not after training alone (Fig. 7D). HSP70 has been shown to play a critical role in the recovery of striated muscle after severe exercise (McArdle et al. 2004) and muscle injury (Oishi et al. 2009). Acute exercise and chronic exercise training for 8 weeks increased HSP70 protein levels in the rat PL (Ogata et al. 2009). Intermittent hyperbaric exposure (3 ATA, 100% O_2_, 1 h daily for 5 consecutive days) increased HSP70 mRNA and protein expression levels in mouse neuroblastoma cells (Shyu et al. 2004). Chronic intermittent hyperbaric exposure used in this study may facilitate the recovery of muscle damage induced by daily exercise via the up‐regulation of HSP70, thereby enhancing muscular adaptation to exercise training.

In conclusion, this study showed that exercise training with intermittent hyperbaric exposure (1.3 ATA with 20.9% O_2_) enhanced endurance performance by promoting oxidative and glycolytic capacities and the expression of proteins involved in mitochondrial biogenesis in mice. Further investigation must be performed to determine whether the present strategy facilitates endurance performance in humans.

## Conflict of Interests

None declared.
